# Respiratory syncytial virus infection in patients with haematological diseases: a retrospective multicentre study

**DOI:** 10.1007/s15010-024-02449-w

**Published:** 2024-12-17

**Authors:** Sebastian Herrmann, Stephanie Graefe, Maximilian Christopeit, Piet Sonnemann, Tessa Hattenhauer, Rebekka Mispelbaum, Malte B. Monin, Hans Martin Orth, Charlotte Flasshove, Henning Gruell, Florian Klein, Uwe Klein, Clara Lehmann, Jan-Hendrik Naendrup, Jannik Stemler, Jon Salmanton-Garcia, Theresa Markus, Oliver A. Cornely, Sibylle C. Mellinghoff

**Affiliations:** 1 Department I of Internal Medicine, European Diamond Excellence Centre for Medical Mycology (ECMM), Centre for Integrated Oncology (CIO), Bonn, Cologne, Düsseldorf, (ABCD), Aachen, Cologne, Germany; 2 Department of Oncology, Haematology and Bone Marrow Transplantation with Section Pneumology, Department of Internal Medicine, University Medical Center Hamburg-Eppendorf, Hamburg, II Germany; 3 Department of Oncology, Haematology, Rheumatology and Immune-Oncology, University Hospital Bonn, Bonn, Germany; 4 Department of Gastroenterology, Hepatology and Infectious Diseases, Medical Faculty, University Hospital Düsseldorf, Düsseldorf, Germany; 5 Institute for Virology, University Hospital Cologne, Cologne, Germany; 6 Institute of Translational Research, Cologne Excellence Cluster on Cellular Stress Responses (CECAD), University of Cologne, Cologne, Germany; 7 German Centre for Infection Research (DZIF), Partner-Site Cologne-Bonn, Braunschweig, Germany; 8 University Cancer Center Hamburg (UCCH), Hamburg, Germany

**Keywords:** RSV, Haematology, Infection prevention, Vaccine

## Abstract

**Purpose:**

This study aims to evaluate the burden of respiratory syncytial virus (RSV) infections in patients with haematological diseases. It seeks to analyse the relevance of prevention, diagnosis and treatment of RSV infections.

**Methods:**

A multi-centre, retrospective study was conducted across University Hospitals in Cologne, Düsseldorf, Bonn, and the University Medical Centre Hamburg-Eppendorf between Jan 2016 and Aug 2023. All haematological patients with diagnosed RSV infection were included. The study focused on the incidence of RSV, underlying conditions, comorbidities, coinfections and clinical outcomes such as hospitalization, intensive care unit (ICU) admission and mortality.

**Results:**

Of 166 patients, 89 (53.6%) had signs of pneumonia and 37 (22.3%) were admitted to ICU due to RSV infection, while 20 (54%) of those were mechanically ventilated with median duration of 11 days (1,33; IQR:18). Mean age was 60 years (range 14–88). Sixteen patients (9.6%) were treated as outpatients, while 52 (31.3%) were hospitalized due to RSV infection; the median hospital stay was 16 days (IQR 25.25, range 0–97). 79 (47.6%) of patients presented with leukopenia and 57 (34.3%) with neutropenia. In total, 22 patients (13.3%) died within 30 days and 29 (17.5%) died within 90 days. Highest mortality rates were seen in patients with aggressive lymphoma (23.5%) and acute leukaemia (18%).

**Conclusion:**

RSV significantly impacts patients with haematological diseases, leading to high rates of hospitalization, ICU admission, and mortality. Preventive measures, such as vaccination, alongside early diagnosis and individualized management, are essential to reduce RSV-associated morbidity and mortality in this high-risk population.

**Supplementary Information:**

The online version contains supplementary material available at 10.1007/s15010-024-02449-w.

## Background

Lower respiratory tract infections (LRTI) are the fourth most common cause of death worldwide with respiratory syncytial virus (RSV) being one of the most common causative viral pathogens [1–3]. RSV is a single-stranded RNA virus from the Pneumoviridae family (Genus Orthopneumovirus). The two subtypes are RSV A and RSV B [4]. RSV can infect cells of the upper and lower respiratory tract and induce the formation of syncytia through cell fusion. LRTI is characterized by inflammation and bronchiolar epithelial necrosis and can result in small airway obstruction [5]. Although RSV infection can affect individuals of all ages, newborns, infants and older adults are at particular risk of severe disease [6].

Despite growing awareness, RSV infections in adult patients may be underestimated. A recent meta-analysis reports approximately 5.2 million RSV cases, 470,000 hospitalizations, and 33,000 in-hospital deaths in ≥ 60-year-old adults in high-income countries annually [7]. Current data estimate 6,000 to 10,000 RSV-related deaths among patients in the U.S. annually [8]. There is no established treatment option and therefore, the development of a vaccine has been prioritized in recent years as key strategy to reducing RSV-associated morbidity and mortality. Since 2023, three vaccines (RSVPreF3 OA, RSVpreF, mRNA1345) against RSV have been licensed in the EU for people above 60 years of age [9–11].

The underestimation is especially pronounced in studies from the cohort of adults with haematologic diseases, where data remains sparse. Existing studies are often constrained by small sample size with the majority focussing on hematopoietic stem cell transplantation [12–16]. The incidence of RSV infections in haematological patients in previous studies varied broadly between 3% and 37%. A recent study of Myeloma (MM) patients showed that the incidence of RSV infections was much higher (16-fold) in Patients with MM compared to the control group [17]. Recent studies have shown mortality rates in haematological patients between 8% and 17% [18]. While the incidence of RSV infections has decreased in 2021/2022 during the Covid-19 pandemic [19], this retrospective, descriptive study intends to provide an comprehensive overview of the aforementioned patient population. It seeks to analyse the relevance of prevention, diagnosis and treatment of RSV infections in patients with haematological diseases, while also raising awareness of the importance of RSV infections in this vulnerable patient population.

## Materials/methods

This retrospective analysis is a multi-centre study performed at the University Hospitals Cologne, Düsseldorf, and Bonn, as well as at the University Medical Centre Hamburg-Eppendorf. The analysis was approved by all institutional review boards (Cologne number 23-1300-retro) and the study was conducted in accordance with Good Clinical Practice (GCP) guidelines to ensure integrity and reliability of the research findings.

All RSV infections confirmed by molecular testing from 01/01/2016 until 08/31/2023 were included. The datasets were provided by the corresponding virologic laboratories, containing patient age, sex and date of RSV diagnosis.

Patients without underlying haematologic malignancy were excluded from the study. Since the RSV vaccine had not yet been approved at the time of the study, no patient was vaccinated against RSV. The electronic patient files from the hospital’s internal documentation system were reviewed to record the following parameters: underlying haematologic malignancy, haematologic malignancy treatment line, leukopenia, neutropenia; symptoms of infection (cough, fever, dyspnoea), presence of pneumonia, hospitalization due to RSV infection and duration of hospitalization, treatment in the intensive care unit (ICU), time on mechanical ventilation, cardiopulmonary comorbidities, viral and bacterial coinfections, survival at days 30 and 90.

Pneumonia was defined as new pulmonary infiltrations in chest computed tomography (CT) scan or X-ray with at least one of the following signs: cough, fever, leucocytosis and pathologic auscultation.

Clinical material for RSV detection were naso- and/or oropharyngeal swabs, tracheal secretions, or bronchial lavages. Diagnosis was made by RT-PCR.

The 14-day period prior to the detection of RSV infection was assessed for leukopenia and neutropenia. Leukopenia was defined as a leucocyte count of less than 4 × 10^9^ per litre, neutropenia as a neutrophil count of less than 0.5 × 10^9^ per litre. Bacterial, fungal and viral coinfections were documented. Patients were screened for the following cardiopulmonary comorbidities: Arterial hypertension, atrial fibrillation, coronary heart disease, myocardial infarction, peripheral arterial occlusive disease, valvular insufficiency, chronic obstructive pulmonary disease (COPD), pulmonary hypertension and malignant lung diseases, bronchopulmonary dysplasia, interstitial lung disease, cystic pulmonary fibrosis, congenital airway anomalies, and asthma. Hospitalization due to RSV infection was considered if the RSV infection was reported as main diagnosis. Patient history was screened for the following clinical symptoms: Cough (clinical history), dyspnoea (clinical history) and fever (> 38.5 °C). If medical history and documentation were inconclusive, absence of data was recorded. The underlying haematological diseases are summarized in the following subgroups: Indolent lymphoma, aggressive lymphoma including diffuse large B-cell lymphoma (DLBCL) and Hodgkin lymphoma (HL), chronic lymphocytic leukaemia (CLL), plasma cell diseases including Myeloma, acute leukaemia including acute myeloid and acute lymphocytic leukaemia (AML, ALL) and myeloproliferative diseases like chronic myelogenous leukaemia (CML). This retrospective study received consent by the local institutional review board (Nr 23-1300-retro).

### Statistical analyses

The relative frequency, mean, median, minimum, maximum, and standard deviation were calculated for all quantitative characteristics. For subgroup analysis, Pearson’s Chi-squared test or Fisher’s exact test was used for categorical variables and t-test or Wilcoxon rank sum test was used for continuous variables. Logistic regression and a logistic regression model were employed to evaluate the effects of various clinical factors on binary outcomes, such as mortality and ICU admission. Kaplan-Meier survival analysis and the log-rank test were used to compare survival between groups. All analyses were performed using SPSS^®^. Visualizations were created in R version 4.30 (R Core Team, 2023) using ggplot2 package.

## Results

**Fig. 1:**
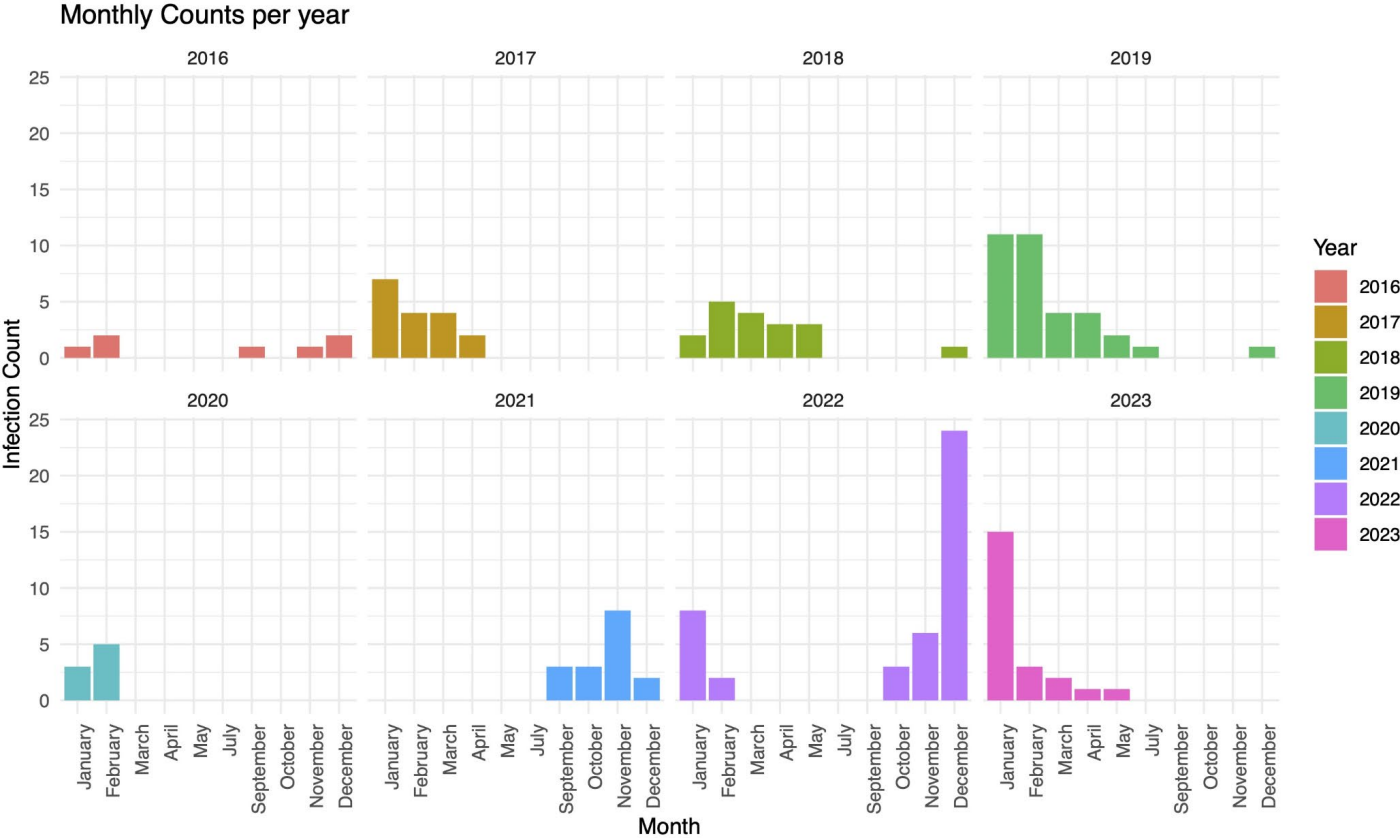
Prevalence of RSV Infection in haematological patients of four German university hospitals from 2016 to 2023

In the 8-year period, 166 patients were included. Most were diagnosed between November and February with peaks in year 2022 and 2023. None were diagnosed in June and August (Fig. 1). Patient characteristics are depicted in Tables 1, 2 and 3.

**Table 1:** Patient characteristics

	Count or Mean (min, max)^1^	% of *n* = 166
**Age**	60 (14, 88; SD 10)	
**Sex**		
Male	104 [17]	62.7
Female	62 [5]	37.3
**Underlying Malignancy**		
Acute leukaemia	51 [9]	30.7
Aggressive lymphoma	34 [8]	20.5
Indolent lymphoma	22 [5]	13.3
Myelodysplastic disease	7 [1]	4.2
Myeloproliferative disease	14 [1]	8.4
Plasma cell disease	32 [2]	19.3
Premalignant and other	6 [0]	3.6
**Chemotherapy Treatment**		
1 Line of Treatment	64 [8]	38.6
2 Lines of Treatment	36 [6]	21.7
≥ 3 Lines of Treatment	47 [5]	28.3
**Leukopenia**		
Yes	79 [17]	47.6
No	75 [5]	45.2
Unknown	12 [0]	7.2
**Neutropenia**		
Yes	57 [15]	34.3
No	89 [6]	53.6
Unknown	20 [1]	12.0
**SCT (Stem Cell Transplant)**		
Allogenic-SCT	44 [5]	26.5
Autologous-SCT	26 [4]	15.7
**Pneumonia**		
Yes	89 [20]	53.6
No	62 [2]	37.3
**Cardiovascular Comorbidities**		
Arterial hypertension	47	28.3
Arrhythmias	28	16.9
Coronary artery disease	20	12.0
Heart valve diseases	12	7.2
Congestive heart failure	9	5.4
Diabetes	10	6.0
Renal diseases (AKI, CKD)	10	6.0
COPD	6	3.6
Peripheral arterial disease	5	3.0
Heart attack	3	1.8

^1^ Patients died after 30-days depicted in brackets []

**Table 2:** Coinfections

	Count^1^	% of *n* = 122 [*n* = 166]
Total	122 [80]	100 [48.2]
Viral	70 [57]	57.3 [34.8]
Adenovirus	3	
CMV	9	
Bocavirus	2	
EBV	10	
HSV	9	
Influenza	5	
Metapneumovirus	4	
Parainfluenza	2	
Parvovirus B19	1	
Rhinovirus	12	
SARS-CoV-2	12	
VZV	1	
Bacterial	33 [29]	27.0 [17.5]
*Acinetobacter pitii*	1	
*Citrobacter koseri*	1	
*Enterobacter cloacae*	1	
*Enterococcus faecium*	4	
*Escherichia coli*	7	
*Haemophilus influenzae*	1	
*Mycobacterium tuberculosis*	1	
*Pseudomonas aeruginosa*	6	
*Serratia marcescens*	1	
*Staphylococcus aureus*	4	
*Staphylococcus epidermidis*	5	
*Streptococcus pneumoniae*	1	
Fungal	19 [17]	15.6 [10.4]
*C. albicans*	5	
*Aspergillus* spp.	12	
*Rhizopus microsporus*	1	
*Pneumocystis jirovecii*	1	

^1^ Number of Patients with Infection depicted in brackets []

**Table 3:** Outcome

	Count/Median (range, IQR)	% of *n* = 166
Hospitalization		
Initially hospitalized due to RSV	52	31.3
Non-hospitalized	16	9.6
Days of Hospitalization	16 (0, 97; IQR: 25)	
**ICU**		
Admission	37	22.3
Pat. with Pneumonia	32	86.5^1^
Pat. with Neutropenia	21	56.8^1^
30-day mortality	18	48.6^1^
90-day mortality	23	62.5^1^
**Mechanical Ventilation**		
Mechanically Ventilated	20	12.0
Days of Mechanical Ventilation	11 (1, 33; IQR: 18)	
**Outcome**		
Day 30	22	13.3
Day 90	29	17.5

^1^ of patients admitted to ICU

Mean age was 60 years (range 14–88). Of the 166 patients, 62 (37.3%) were female. The most common underlying haematological disease in our cohort was acute leukaemia in 51 (30.7%). Other common underlying diseases included plasma cell diseases (32 [19.3%]), indolent lymphoma (21 [12.7%]), and aggressive lymphoma (17 [10.2%]). Twenty-six patients had undergone autologous stem cell transplantation (SCT) and 44 allogeneic SCT prior to RSV infection. Seventy-nine (47.6%) patients had leukopenia and 57 (34.3%) neutropenia when infected with RSV.

Sixteen patients (9.6%) were treated as outpatients, while 52 (31.3%) were hospitalized because of RSV infection and in 88 patients (53.0%) RSV infections occurred during hospitalisation. The median hospital stay was 16 days (IQR 25.25, range 0–97). Symptoms reported included cough in 102 (61.4%), dyspnoea in 70 (39.8%), and fever in 74 (44.6%). More than half of the patients (53.6%) had pneumonia and in 37 (22.3%) RSV infection led to intensive care treatment, while 20 (54%) were mechanically ventilated with a median duration of 11 days (1.33; IQR:18).

Of the patients, 104 (62.7%) had pre-existing cardiopulmonary comorbidities. Most common were arterial hypertension in 44 (28.3%), arrhythmias in 28 (16.9%), and coronary artery disease in 20 (12.0%).

In our patient cohort, coinfections were present in 48.2% of cases (80), with viral infections being the most common (57[34.8%]), followed by bacterial coinfections (29[17.5%]) and fungal coinfections (19[10.4%]). Viral coinfections were most commonly caused by respiratory viruses (37 of 70 viral coinfections), while bacterial co-infections were most frequently caused by gram-negative pathogens (22 of 33). Invasive fungal co-infections were candidemia (*n* = 5), invasive pulmonary aspergillosis (*n* = 12 probable, 2 possible), and by *Rhizopus microsporus* and *Pneumocystis jiroveci*, one each.

In total, 22 patients (13.3%) died within 30 days and an additional 7 (4.2%) died within 90 days. Highest mortality rates were seen in patients with aggressive lymphoma (23.5%) and acute leukaemia (18%).

**Fig. 2:**
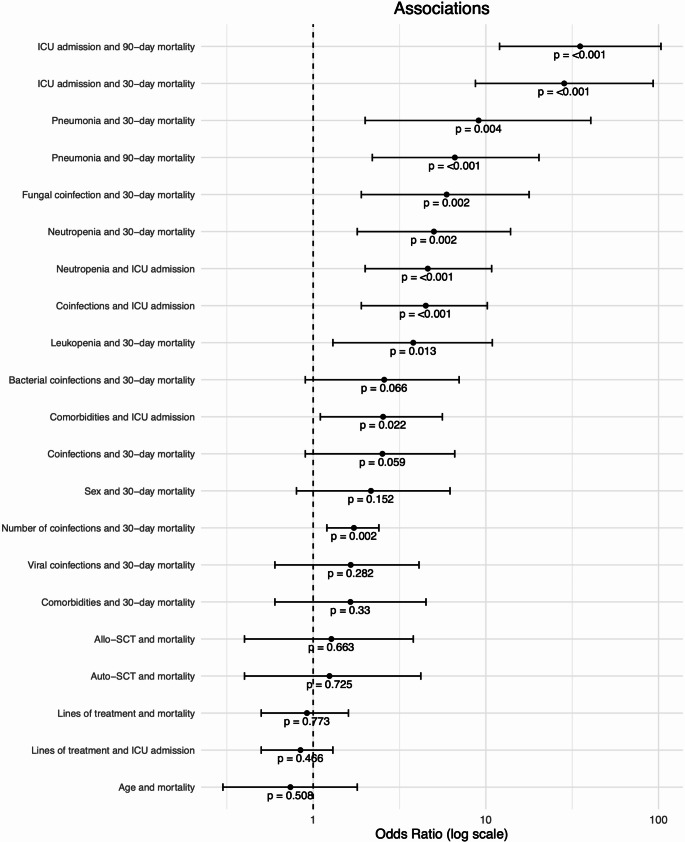
Association of clinical parameters with mortality

**Fig. 3:**
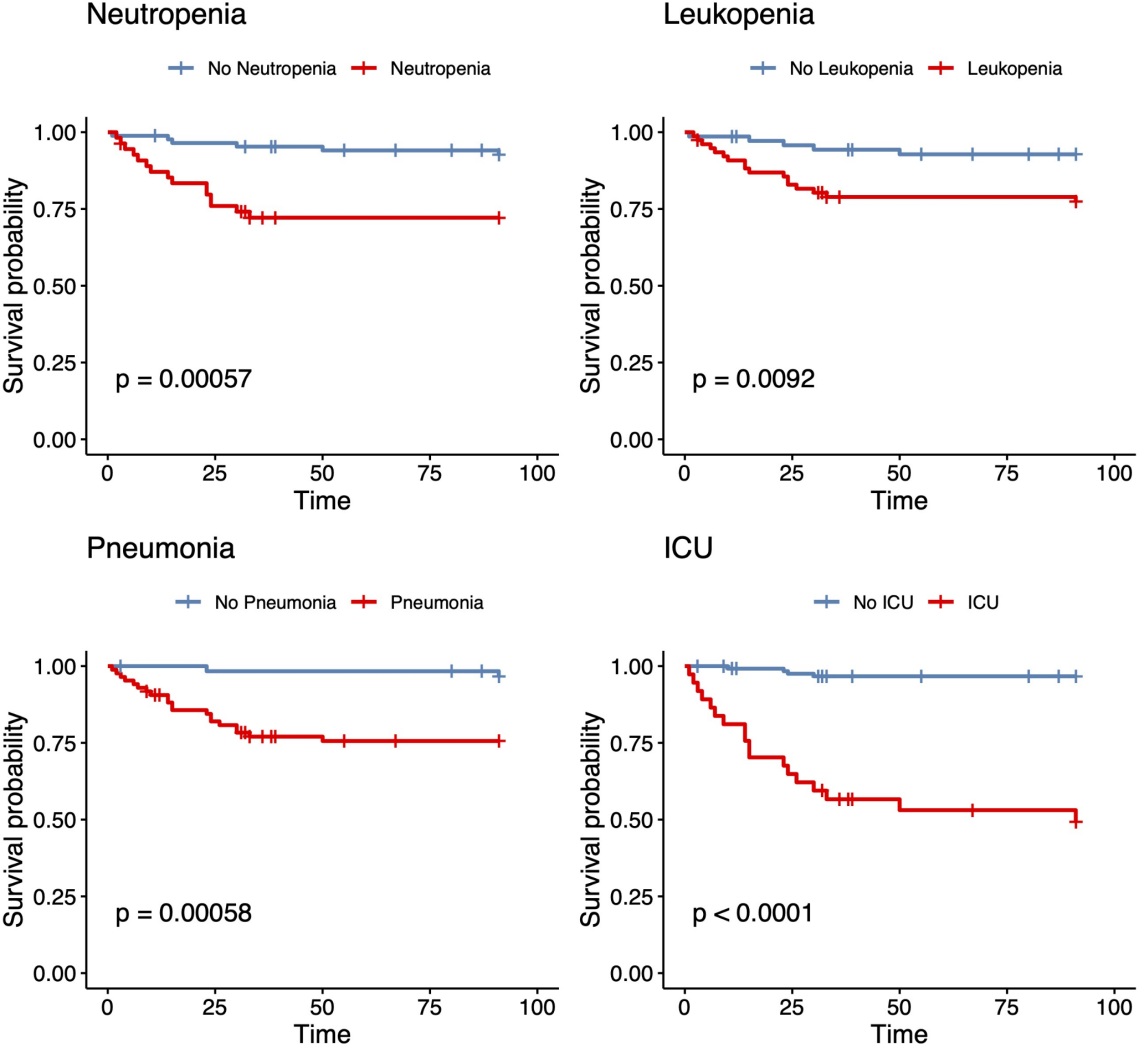
Kaplan Meier survival rates

Neutropenia (OR 5.0, 95% CI 1.8–13.9, *p* = 0.002) and leukopenia (OR 3.8, 95% CI 1.3–10.9, *p* = 0.013) were linked to increased likelihood of ICU admission (neutropenia OR 4.6, 95% CI 2.0-10.8, *p* = 0.001; leukopenia OR 2.5, 95% CI 1.1–5.6, *p* = 0.022). Additionally, both were significantly associated with a higher 30-day mortality rate in the univariate analysis (Figs. 2 and 3).

Thirty-two (86.5%) patients admitted to the ICU were diagnosed with pneumonia, compared to 57 (44.2%) of those not admitted to the ICU (OR 8.1, 95% CI 2.7–24.5, *p* = 0.001). Admission to the ICU was significantly associated with increased mortality, with 48.6% of ICU patients dying within 30 days (OR 28.4, 95% CI 8.7–93.1, *p* = 0.001) and 62.5% within 90 days (OR 35.1, 95% CI 12.0-103.3, *p* = 0.001).

No significant effect of comorbidities, including cardiovascular, lung, or renal diseases, on mortality was observed in the univariate regression analysis (OR 1.643, 95% CI 0.6–4.5, *p* = 0.330). Patients with coinfections, neutropenia, and pneumonia had longer hospital stays, respectively.

Coinfections were associated with a higher likelihood of ICU admission (OR 4.487, 95% CI 1.9–10.2, *p* < 0.001). Also, a significant association was observed between the number of coinfections and the mortality rate (OR 1.721, 95%CI 1.2–2.4, *p* = 0,002) with fungal coinfections particularly increasing mortality risk (OR 5.927, 95% CI 1.9–17.8, *p* = 0.002).

### Multivariate analysis

A logistic regression was performed to ascertain the effects of the factors age, sex, leukopenia, neutropenia, comorbidities and coinfections on mortality. Only Neutropenia showed significant impact on the outcome in this model. Neutropenic patients with RSV were five times more likely to die than non-neutropenic patients (OR 4.917, *p* = 0.004). The logistic regression model was statistically significant (χ^2^(5) = 17.392, *p* < 0.004) and explained 21% (Nagelkerke R2) of the variance in mortality.

To further investigate the impact of coinfections we built a second model including Coinfections divided into subgroups viral, bacterial and fungal. In this second model fungal coinfections (OR 4.248, *p* = 0.033) and neutropenia (OR 4.673, *p* = 0.008) showed a significant impact on patients’ outcome (χ^2^(7) = 23.773, *p* < 0.001). The model explained 27% (Nagelkerke R2) of the variance in mortality.

## Discussion

We analysed clinical characteristics and outcomes of patients with haematological malignancies and RSV infection at four German centres from January 2016 to August 2023. We report risk factors and clinical outcomes associated with RSV infections, providing insights into the prognosis of these vulnerable patients.

RSV infection is a common cause of LRTI in adult patients with haematological malignancies. Data on the incidence of RSV among these patients vary widely from 5 to 49% [18, 20–24], while representing approximately 30 to 37% [25, 26] of all respiratory viral infections. Diagnostic methods and testing frequency impact reported incidences as well as seasonality and geography [27, 28]. We confirm seasonal occurrence in our cohort from November to February. Furthermore, an absence of RSV infections between March 2020 and July 2021 was observed underlining the significant impact of Covid-19 pandemic-related prophylactic measures on other respiratory viruses like RSV.

Our cohort reflects an adult patient population at risk for RSV-associated disease with lower mean age than reported in immunocompetent cohorts [2]. While other studies indicate older age and male sex as risk factors for worse outcomes in RSV infections, we only observed trends towards severe disease course as well as worse outcome in these groups. This mirrors a more decisive role of other risk factors, such as comorbidities, antineoplastic treatment and associated neutropenia as well as the haematological condition per se. The significant association between neutropenia and leukopenia with higher 30-day mortality aligns with other studies [25, 29] indicating that immunocompromised patients, particularly those with haematological malignancies, are at increased risk for worse outcome of RSV infection. In a previous study [30] neutropenia was also identified as a major risk factor for severe RSV infection, noting that it contributes to prolonged viral shedding and a higher likelihood of ICU admission. In addition, it poses patients at high risk for bacteraemia and fungal infections as reported in our cohort, in turn increasing morbidity and associated mortality. As a predictor of poor outcomes, these observations further reinforce the importance of prevention-measures in the neutropenic patient population, in particular when managed with increasing frequency as outpatients.

The correlation between pneumonia, ICU admission, and increased mortality is consistent with findings from other studies on RSV, which identify lower respiratory tract involvement as a critical factor in determining a severe disease course. A meta-analysis focusing on patients following stem cell transplantation reported similar associations, where RSV-related pneumonia was linked to higher ICU admissions and mortality, particularly in those vulnerable subgroups with underlying respiratory conditions or immunosuppression [13]. The observation that allogeneic or autologous SCT are not significantly associated with higher mortality contrasts with some reports that suggest transplant recipients are at elevated risk for severe RSV infection [30]. This discrepancy might be due to differences in study populations or advancements in prophylactic strategies in transplant recipients during the COVID-19 pandemic. However, the association between SCT and higher hospitalization rates is consistent with the known vulnerability of these patients to respiratory infections, including RSV.

In 2023 and 2024, three RSV vaccines received approval for the European market by the European Medicines Agency (EMA), marking a significant advancement in the management of RSV, particularly for vulnerable populations [9–11]. The pivotal trials for these vaccines investigated the prevention of symptomatic RSV-associated LRTI. Of note, the available phase III studies systematically excluded immunocompromised patients and data on vaccine efficacy and safety are hence lacking in this population. Given that neutropenia and leukopenia are strongly associated with increased mortality and ICU admissions, as confirmed by our data, preventing RSV infection through vaccination could be particularly beneficial for this high-risk group. By preventing the initial infection or reducing its severity, vaccines could significantly decrease the likelihood of complications such as pneumonia, which is a major driver of mortality in the studied cohort. The high rate of ICU admissions among patients with severe RSV, particularly those with pneumonia, suggests that effective vaccination could alleviate the burden on healthcare systems. Reducing the number of severe RSV cases through vaccination may have the potential to lower ICU admission, shorten hospital stay, and decrease mortality within the cohort of immunocompromised patients. Our findings underscore the critical need for adequate prevention of RSV, especially among high-risk groups and including these into prospective vaccine trials. While immunocompromised patients may have a suboptimal immune response to vaccines due to their underlying conditions or treatments, even partial protection could reduce the severity of RSV infections. As a consequence, the *Ständige Impfkommission* (STIKO) has just recommended the vaccination of patients aged over 60 and those with pre-existing conditions [31]. Additionally, the *Deutsche Gesellschaft für Hämatologie und Medizinische Onkologie* (DGHO) and the *Deutsche Gesellschaft für Pneumologie und Beatmungsmedizin* (DGP) have recently recommended vaccination in adults [32]. Future trials should focus on analysing vaccine-induced immunity to identify the best vaccines and optimize vaccination schedules, including potential booster vaccinations.

This study has several limitations. Its retrospective nature does limit the generalizability of our findings and causality between RSV detection and outcomes cannot be established. Further, coinfections, might be over- or underrepresented due to incomplete or inconsistent documentation. An inherent diagnostic selection bias limits findings to mainly symptomatic and hospitalized patients and excludes incidence and the course of disease in the outpatient setting. A presumably high number of outpatient cases with a mild course is conceivable. In addition, an RSV-negative control group is lacking and therefore, severe cases may be overreported. An RSV-negative control group helps isolate RSV’s specific impact on disease severity, enabling accurate comparisons and minimizing bias from confounding factors. While coinfections were linked with higher ICU admission rates and mortality, it remains to be determined whether these infections directly contributed to worse outcomes or if severe RSV predisposed patients to secondary infections. Moreover, we were unable to control for all potential confounders, such as the timing and severity of individual infections or the use of antimicrobial therapy which could also influence patient outcomes. The sample size, while adequate for preliminary insights, may not fully capture the diversity of the population. Future research should address these limitations by employing prospective designs and larger, even more representative samples to validate and extend these findings while rapid antigen tests may be an additional tool to effectively screen outpatients for RSV infections [33].

Despite the acknowledged limitations, this multicentre study underscores that respiratory syncytial virus (RSV) represents a significant and prevalent infection in patients with haematologic malignancies, often associated with severe clinical outcomes. Consequently, integrating emerging RSV vaccines into the preventive measures for immunocompromised individuals, particularly those with haematological malignancies, holds the potential to markedly improve patient outcomes by reducing both the incidence and severity of RSV-related illness. Such preventive strategies could lead to a decrease in ICU admissions, mitigate the occurrence of severe complications such as pneumonia and coinfections, and ultimately lower mortality rates. Therefore, we strongly advocate for the inclusion of vulnerable patient populations, frequently excluded from large-scale clinical trials, in future RSV vaccine studies. This approach is essential for advancing the level of evidence in this critical area of patient care.

## Electronic supplementary material

Below is the link to the electronic supplementary material.

Supplementary Material 1

## Data Availability

Data will be made available upon request to the corresponding author.
