# Supplementary material for: Respiratory syncytial virus infection in patients with haematological diseases: a retrospective multicentre study
Source: Infection. 2024 Dec 17;53(4):1341–50. doi: 10.1007/s15010-024-02449-w (PMC12316825; doi:10.1007/s15010-024-02449-w)
Supplement: Supplementary file 1 — Supplementary Material 1 [file 15010_2024_2449_MOESM1_ESM.docx]

**Supplementary material for Respiratory Syncytial Virus Infection in Patients with Haematological Diseases: A retrospective multicentre study**

Sebastian Herrmann^1^, Stephanie Graefe^2^, Maximilian Christopeit^2^, Piet Sonnemann^2^ Tessa Hattenhauer^3^, Rebekka Mispelbaum^3^, Malte B Monin^4^, Hans Martin Orth^5^, Charlotte Flasshove^5^, Henning Gruell^6^, Florian Klein^6^, Uwe Klein^4^, Clara Lehmann^1^, Jan-Hendrik Naendrup^1^, Jannik Stemler^1,7,8^, Jon Salmanton-Garcia^7,8^, Theresa Markus^1^, Oliver A. Cornely^1,7,8^, Sibylle C. Mellinghoff^1,7,8^

1 Department I of Internal Medicine, European Diamond Excellence Centre for Medical Mycology (ECMM), and Centre for Integrated Oncology (CIO), Aachen, Bonn, Cologne, Düsseldorf, (ABCD), Cologne, Germany

2 Department of Oncology, Haematology and Bone Marrow Transplantation with Section Pneumology, II. Department of Internal Medicine.

3 Department of Oncology, Haematology, Rheumatology and Immune-Oncology, University Hospital Bonn, Bonn, Germany

4 Department of Oncology, Haematology, Rheumatology and Immune-Oncology, University Hospital Bonn, Bonn, Germany

5 Department of Gastroenterology, Hepatology and Infectious Diseases, Medical Faculty and University Hospital Düsseldorf, Düsseldorf, Germany

6 Institute for Virology, University Hospital Cologne, Cologne, Germany

7 Institute of Translational Research, Cologne Excellence Cluster on Cellular Stress Responses (CECAD), University of Cologne, Cologne, Germany

8 German Centre for Infection Research (DZIF), Partner-Site Cologne-Bonn

**Corresponding author**: PD Dr. med. Sibylle C. Mellinghoff; [Sibylle.mellinghoff@uk-koeln.de](mailto:Sibylle.mellinghoff@uk-koeln.de)

| **Association** | **Chi square analysis** | | | **Univariate regression analysis** | | | **(1) Multivariate analysis** | | | **(2) Multivariate analysis** | | |
| --- | --- | --- | --- | --- | --- | --- | --- | --- | --- | --- | --- | --- |
|  | **Chi^2** | **df** | **p-value** | **Odds Ratio** | **95% (CI)** | **p-value** | **OR** | **95% (CI)** | **p-value** | **OR** | **95% CI** | **p-value** |
| Neutropenia and 30-day mortality | 10.902 | 1 | <0.001 | 5.000 | 1.8-13.9 | 0.002 | 4.917 | 1.6-14.6 | 0.004 | 4.673 | 1.5-14.6 | 0.008 |
| Neutropenia and ICU admission | 13.622 | 1 | <0.001 | 4.608 | 2.0-10.8 | <0.001 | - | - | - | - | - | - |
| Leukopenia and 30-day mortality | 6.771 | 1 | 0.009 | 3.797 | 1.3-10.9 | 0.013 | - | - | - | - | - | - |
| Leukopenia and ICU admission | 5.409 | 1 | 0.020 | 2.539 | 1.1-5.6 | 0.022 | - | - | - | - | - | - |
| Pneumonia and 30-day mortality | 14.963 | 2 | <0.001 | 9.077 | 2.0-40.5 | 0.004 | - | - | - | - | - | - |
| Pneumonia and 90-day mortality | 18.164 | 2 | <0.001 | 6.611 | 2.2-20.3 | <0.001 | - | - | - | - | - | - |
| Pneumonia and ICU admission | 20.688 | 2 | <0.001 | 8.140 | 2.7-24.5 | <0.001 | - | - | - | - | - | - |
| ICU admission and 30-day mortality | 49.838 | 1 | <0.001 | 28.421 | 8.7-93.1 | <0.001 | - | - | - | - | - | - |
| ICU admission and 90-day mortality | 63.512 | 1 | <0.001 | 35.139 | 12.0-103.3 | <0.001 | - | - | - | - | - | - |
| Sex and 30-day mortality | 2.126 | 1 | 0.145 | 2.160 | 0.8-6.2 | 0.152 | 2.672 | 0.8-8.9 | 0.112 | 2.358 | 0.7-8.2 | 0.178 |
| Coinfections and ICU admission | 13.833 | 1 | <0.001 | 4.487 | 1.9-10.2 | <0.001 | - | - | - | - | - | - |
| Coinfections and 30-day mortality | 3.737 | 1 | 0.053 | 2.517 | 0.9-6.6 | 0.059 | 2.107 | 0.7-6.4 | 0.188 | - | - | - |
| Viral coinfections and 30-day mortality | 1.172 | 1 | 0.279 | 1.649 | 0.6-4.1 | 0.282 | - | - | - | 1.693 | 0.6-5.0 | 0.346 |
| Bacterial coinfections and 30-day mortality | 3.553 | 1 | 0.059 | 2.578 | 0.9-7.0 | 0.066 | - | - | - | 2.584 | 0.8-8.1 | 0.105 |
| Fungal coinfections and 30-day mortality | 11.935 | 1 | <0.001 | 5.927 | 1.9-17.8 | 0.002 | - | - | - | 4.248 | 1.1-16-0 | 0.033 |
| Number Coinfections and 30-day mortality | 21.332 | 6 | 0.002 | 1.721 | 1.2-2.4 | 0.002 | - | - | - | - | - | - |
| Comorbidities and 30-day mortality | 0.964 | 1 | 0.326 | 1.643 | 0.6-4.5 | 0.330 | 2.185 | 0.6-8.0 | 0.237 | 1.920 | 0.5-7.3 | 0.340 |
| Age and mortality | 0.441 | 1 | 0.507 | 0.738 | 0.3-1.8 | 0.508 | 1.005 | 0.9-1.0 | 0.807 | 1.017 | 0.9-1.1 | 0.476 |
| Allo-SCT and mortality | 0.272 | 1 | 0.602 | 1.274 | 0.4-3.8 | 0.663 | - | - | - | - | - | - |
| Auto-SCT and mortality | 0.215 | 1 | 0.643 | 1.243 | 0.4-4.2 | 0.725 | - | - | - | - | - | - |
| Comorbidities and ICU admission | 1.181 | 1 | 0.277 | 2.539 | 1.1-5.6 | 0.022 | - | - | - | - | - | - |
| Lines of treatment and ICU admission | 1.418 | 2 | 0.492 | 0.844 | 0.5-1.3 | 0.466 | - | - | - | - | - | - |
| Lines of treatment and 30-day mortality | 0.592 | 2 | 0.744 | 0.920 | 0.5-1.6 | 0.773 | - | - | - | - | - | - |
| Haem. malignancy and 30-day mortality | 9.275 | 9 | 0.412 | - | - | - | - | - | - | - | - | - |
| **Association** | **Wilcoxon** | | |  |  |  | (1) (χ2(5) = 17.392, p <0.004) | | | (2) (χ2(7) = 23.773, p <0.001) | | |
| Hosp. stay and Coinfections | U=2086 | Z= -3.805 | <0.001 | - | - | - | - | | | - | - | - |
| Hosp. stay and Neutropenia | U=1587.5 | Z=-4.377 | <0.001 | - | - | - | - | | | - | - | - |
| Hosp. stay and Pneumonia | U = 1520 | Z=-4.377 | <0.001 | - | - | - | - | | | - | - | - |

**Supplemental Table 1** *Univariate and multivariate analysis*
